# Locating Damage Using Integrated Global-Local Approach with Wireless Sensing System and Single-Chip Impedance Measurement Device

**DOI:** 10.1155/2014/729027

**Published:** 2014-01-06

**Authors:** Tzu-Hsuan Lin, Yung-Chi Lu, Shih-Lin Hung

**Affiliations:** ^1^Sinotech Engineering Consultants, Inc., No. 280, Xinhu 2nd Road, Neihu Distict, Taipei 11494, Taiwan; ^2^Department of Civil Engineering, National Chiao Tung University, 1001 Ta Hsueh Road, Hsinchu 300, Taiwan

## Abstract

This study developed an integrated global-local approach for locating damage on building structures. A damage detection approach with a novel embedded frequency response function damage index (NEFDI) was proposed and embedded in the Imote2.NET-based wireless structural health monitoring (SHM) system to locate global damage. Local damage is then identified using an electromechanical impedance- (EMI-) based damage detection method. The electromechanical impedance was measured using a single-chip impedance measurement device which has the advantages of small size, low cost, and portability. The feasibility of the proposed damage detection scheme was studied with reference to a numerical example of a six-storey shear plane frame structure and a small-scale experimental steel frame. Numerical and experimental analysis using the integrated global-local SHM approach reveals that, after NEFDI indicates the approximate location of a damaged area, the EMI-based damage detection approach can then identify the detailed damage location in the structure of the building.

## 1. Introduction

Monitoring the health of a structural system depends on an effective damage identification process that provides the location, determination of type, and estimation of severity of the damage caused. Damage is defined as a change to a structural system, for example, its material and/or geometric properties that affects its current or future performance [1–4]. Structural health monitoring (SHM) is broadly classified into global and local methods. Global SHM can identify damage that affects the overall structure or large portions of the structure.

Most available damage detection methods identify damage by measuring changes in frequency mode shape, curvature of mode shape; dynamically measuring flexibility, and updating structural model parameters that are extracted from structural responses that are measured using low-cost sensing systems [5]. Cawley and Adams [6] utilized frequency shifts to detect damage in composite materials, by considering the ratio between the frequency shifts of two modes, and considered a number of mode pairs to identify potential damage locations with the lowest error. However, their method was ineffective when damage had occurred at multiple locations. Pandey et al. [7] established a central difference operator-based method of using absolute changes in mode shape curvature to locate structural damage in FEM beams. The mode shape curvature-based method is more sensitive than the mode shape-based method. Global SHM methods have been reviewed elsewhere [5, 8, 9].

Nonparametric global damage detection approaches, such as the frequency response function- (FRF-) based method, are also extensively used in SHM. Thyagarajan et al. [10] developed a method of using FRF data and data from an optimal number of sensors on a structure to locate damage for a structure with many degrees of freedom. Maia et al. [11] proposed an FRF curvature-based damage detection method and compared its performance with that of a conventional mode shape-based method. Their comparison demonstrated that methods based on FRF curvatures outperformed mode shape-based methods. Sampaio et al. [12] developed a theoretical FRF curvature method that used measured FRF and neural networks (NNs), and they evaluated the efficiency of this method by using numerically simulated data and experimental data for an actual bridge. Furukawa et al. [13] developed a damage detection method using uncertain FRFs based on a statistical bootstrap method. Ni et al. [14] identified seismic damage to a 38-storey building model and determined FRFs for dimensionality reduction and noise elimination based on a principal component analysis (PCA). Their results indicated that PCA can feasibly be used to filter unwanted measurement noise. Kanwar et al. [15] demonstrated the feasibility of using FRF to evaluate structural damage to reinforced concrete buildings.

Nondestructive testing (NDT) is used mostly in local damage detection. The numerous NDT techniques include the use of ultrasound, infrared thermography, and Eddy current X-rays. Although such methods can detect minute or incipient damage, they usually require expensive and bulky equipment with high power requirements. The electromechanical impedance- (EMI-) based damage detection method is a new NDT that analyzes the electromechanical coupling property of piezoelectric materials. The EMI-based damage detection method uses active surface-bonded PZT (lead zirconate titanate) patches to sense structural-mechanical changes in impedance. Liang et al. [16] were the first to propose an electrical admittance model of a PZT bar that is connected to a structure. Subsequently, Sun et al. [17] experimentally utilized an electrical impedance method to obtain the FRF for single and multiple locations on a structure. This EMI-based method has proven to be effective in various experimental applications, including aircraft structures [18], temperature-variable applications [19], civil structures [20], concrete structures [21], and PSC girder bridges [22]. Impedance methods also continue to attract the interest of researchers and field engineers. Park et al. [23] summarized unresolved issues associated with the impedance method in the areas of both hardware and software. Such issues include the difficulty in handling crisp PZT sensors and bonding them to the structure, the bulky and expensive analyzers that are required for testing impedance, the difficulty and complexity of acquiring and processing data on large-scale complex structures, and high power consumption.

Typically, sensors are an important part of an SHM process. Recent developments in smart sensor technology support new applications in SHM. The main features of a typical smart sensor are its on-board microprocessor, sensing capability, data storage, wireless communication, battery power, and low cost. The long-term recording of data for monitoring purposes is expensive [24–26]. Smart sensors can process data before outputs are recorded, reducing the amount of data transmitting and computing power required. When numerous sensors are adopted, wireless communication is particularly favorable. Wired sensor systems can only deploy a limited number of sensors because of cost or complexity constraints. Wireless sensors mitigate these problems by simplifying installation. Smart sensor-based wireless sensor networks (WSNs) are attractive for monitoring structural health because of their low manufacture costs, low power requirements, small size, and simplicity of deployment (lack of cables) [27, 28].

Since both local SHM and global SHM have unique capabilities and shortcomings, an integrated approach may be more effective than either method alone. An embedded damage detection approach with a novel embedded FRF damage index (NEFDI) is proposed for detecting damage to an entire structure. Implementing an embedded damage detection algorithm in a complex development environment commonly involves hardware and software complexity and so is quite challenging, especially for civil engineers with limited experience of WSNs. Also, robust system development is problematic in a complex programming environment.

Accordingly, this work develops an easy-to-use development environment that is based on the .NET Micro Framework. Related applications can be implemented efficiently in the .NET Micro Framework. An embedded damage detection approach that uses a novel FRF damage index (NEFDI) is developed, and an NEFDI-based protocol is embedded in the Imote2.NET-based wireless SHM system to locate damage to building structures. This wireless SHM system is based on Imote2.NET, which is an advanced sensor platform that is compatible with the .NET Micro Framework. Local damage is then identified by the EMI-based damage detection method. In this work, electromechanical impedance was acquired using single-chip impedance measurement devices. The feasibility of the proposed damage detection procedure was studied using a numerical example of a six-storey shear plane frame structure that is subjected to base excitation. The proposed damage detection approach was then tested on a small-scale four-storey steel frame. Finally, the proposed approach was used to locate damage to an experimental (1/4)-scale six-storey steel structure.

## 2. Global-Local-Integrated Damage Detection Approach

**Figure 1:**
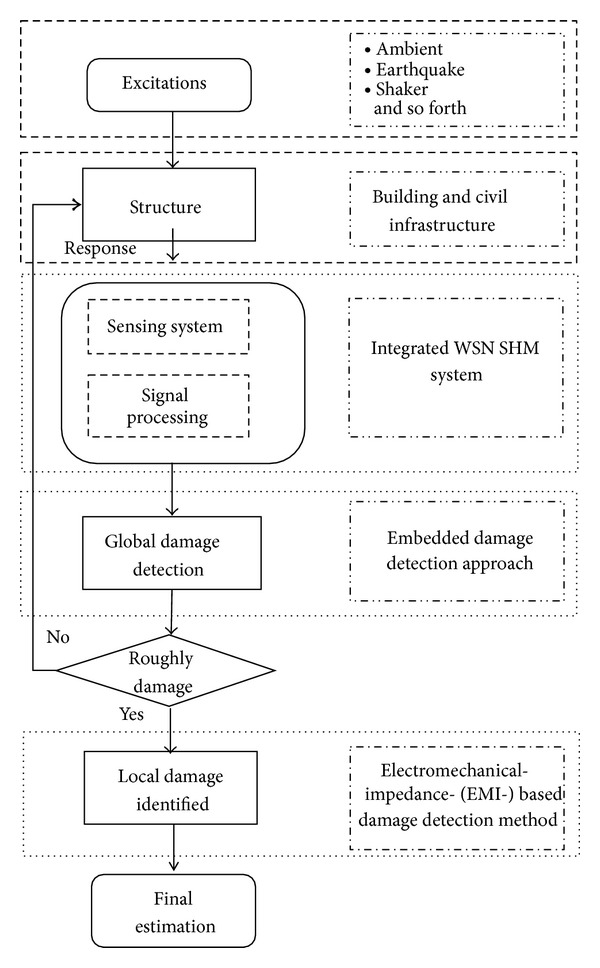
Global-local-integrated damage detection approach.

Figure 1 presents the stages of the proposed integrated global-local damage detection approach. In the first stage, the global approach detects changes in the characteristics of the overall structure. Then, in the second stage, the damage is identified in detail and its approximate location is determined. For instance, in a building structure, global SHM can yield a rough estimate of the location of a damaged floor. Local SHM then checks in detail the components of the structure of the building. A damage detection approach with a novel embedded FRF damage index (NEFDI) is proposed for global damage detection. Finally, local structural damage is detected using an EMI-based method.

## 3. An Embedded Damage Detection Approach for Global Damage Detection

### 3.1. FRF-Based Damage Detection Method

For a damped structure with multiple (*N*) degrees of freedom (MDOF), the equations of motion are

(1) $$\begin{array}{c} \left[M\right]\left\{\ddot{x}\left(t\right)\right\}+\left[C\right]\left\{\dot{x}\left(t\right)\right\}+\left[K\right]\left\{x\left(t\right)\right\}=\left\{f\left(t\right)\right\}, \end{array}$$

where [*M*], [*C*], and [*K*] are *N* × *N* matrices for mass, viscous damping, and stiffness, respectively, and *x*(*t*) and *f*(*t*) are *N* × 1 vectors of the displacement functions and external excitation load, respectively. Applying the Fourier transformation to (1) and performing simple algebraic manipulation yield

(2) $$\begin{array}{c} \left\{X\right\}={\left(\left[K\right]+i\omega \left[C\right]-\omega^{2}\left[M\right]\right)}^{-1}\left\{F\right\}. \end{array}$$

This can be written simply as

(3) $$\begin{array}{c} \left\{X\right\}=\left[H\left(\omega \right)\right]\left\{F\right\}, \end{array}$$

where [*H*(*ω*)] is an FRF of the structure.

The total stiffness and damping matrices, [*K*] and [*C*], of an intact structure are the summations of the corresponding matrices of the elements and are given by

(4) $$\begin{array}{c} \left[K\right]=\sum_{e}^{n}\left[K^{e}\right],\;\;\left[C\right]=\sum_{e}^{n}\left[C^{e}\right], \end{array}$$

where *n* is the number of elements and [*K* ^*e*^] and [*C* ^*e*^] (*e* = 1,…, *n*) are the stiffness and damping of the *e*th element, respectively, contributing to the total stiffness and damping matrices.

Damage is theoretically assumed to reduce [*ηK*] or increase [*βC*]. Variations in total stiffness and damping matrices are calculated by summing the changes in the stiffness and damping matrices of elements:

(5) $$\begin{array}{c} \left[\eta K\right]=\sum_{e}^{n}\left[\eta^{e}K^{e}\right],\;\;\left[\beta C\right]=\sum_{e}^{n}\left[\beta^{e}C^{e}\right], \end{array}$$

where *η* ^*e*^ and *β* ^*e*^ are the proportional changes in stiffness and damping, respectively, of the *e*th element.

By applying the damage matrices, the equation of motion for a damaged structure can be expressed as

(6) [M]({x¨}+{δx¨})+([C]+[βC])({x˙}+{δx˙}) +([K]−[ηK])({x}+{δx})={f(t)},

where {*δx*} is the increase in displacement.

A simple reformulation yields

(7) [M]{δx¨}+([C]+[βC]){δx˙}+([K]−[ηK]){δx}  =([ηK]{x}−[βC]{x˙}).

Applying the Fourier transformation to (7) and substituting (7) in (2) and (3) yield

(8) (−ω2[M]+iω([C]+[βC])+([K]−[ηK])){δX}  =([ηK][H(ω)]−iω[βC][H(ω)]){F}.

Applying (2) to solve (8) yields {*δX*}:

(9) {δX}=([Hδ′(ω)][ηK][H(ω)] −iω[Hδ′(ω)][βC][H(ω)]){F},

where *H*_*δ*_′(*ω*) is the FRF of the damaged structure with respect to {*δx*} and is expressed as

(10) Hδ′(ω)=(−ω2[M]+iω([C]+∑en[βeCe])   +([K]−∑en[ηeKe]))−1.

Based on (10), the change in the FRF is related to damage and can therefore be utilized as a critical index in locating damage to building structures. Initially, sensors are deployed on the shear building and they will measure response data immediately during an earthquake excitation. The initial stiffness of the structure is defined, and the corresponding FRFs *H*_*u*_^*i*^(*ω*) are computed as a known undamaged state, which is also referred to as a before-scenario state. Thereafter, the sensors continuously collect seismic response data concerning the structure after each earthquake, and the FRFs, *H*_*d*_^*i*^(*ω*), are thus obtained; these describe the after-scenario state. Consider a shear building structure with *N* dof to locate and quantify damage, a novel FRF damage index (NEFDI) is proposed. The NEFDI is an extension of the frequency response function curvature method and is defined as

(11) $$\begin{array}{c} \text{NEFD}{\text{I}}_{i}=2\text{IS}{\text{D}}_{i}-\text{IS}{\text{D}}_{i-1}-\text{IS}{\text{D}}_{i+1}\;i=2,3,\ldots ,N-1. \end{array}$$

If NEFDI < 0, then NEFDI = 0, where the *in situ* damage index, ISD_*i*_, is expressed as

(12) $$\begin{array}{c} \text{IS}{\text{D}}_{i}=\frac{\sum_{\omega =a}^{b}\left\{{\left(\left|H_{d}^{i}\left(\omega \right)\right|-\left|H_{u}^{i}\left(\omega \right)\right|\right)}^{2}\times {\left(D_{\max}\right)}^{-1}\right\}}{n}, \end{array}$$

where *a*, *b*, and *n* are working parameters and *D*_max⁡_ is given by

(13) $$\begin{array}{c} D_{\max}=\max\left[{\left\{\left|H_{d}^{i}\left(\omega \right)\right|\right\}}_{\max},{\left\{\left|H_{u}^{i}\left(\omega \right)\right|\right\}}_{\max}\right]. \end{array}$$

The |*H*_*d*_^*i*^(*ω*)| and |*H*_*u*_^*i*^(*ω*)| are the magnitudes of the FRF for the *i*th dof of the structure in damaged and undamaged states, respectively. The range of selected frequencies for calculating NEFDI_*i*_ is set to *a*–*b*, where *a* is the starting frequency and *b* is the end frequency.

Notably, when *i* = 1 or *N*, referring to the first or last dof of the structural system, respectively, the NEFDI is given by

(14) $$\begin{array}{c} \text{NEFD}{\text{I}}_{1}=2\left(\text{IS}{\text{D}}_{1}-\text{IS}{\text{D}}_{2}\right),\\ \text{NEFD}{\text{I}}_{N}=2\left(\text{IS}{\text{D}}_{N}-\text{IS}{\text{D}}_{N-1}\right). \end{array}$$

### 3.2. Numerical Study

A numerical example of a six-storey plane frame structure (simulated using SAP2000) that is subjected to base excitation is utilized to evaluate the feasibility of the proposed damage detection scheme. All floors had the same structural parameters—mass *m*_*i*_ = 30 kg and stiffness *k*_*i*_ = 55.5 kN/m (*i* = 1–6). A numerical simulation was carried out with 5% modal damping. The damage was simulated as reduced storey stiffness. Dam_*k*_*i*_ (*i* = 1–6) denotes damage in the form of reduced stiffness *k*_*i*_ at a single site *i*; similarly, Dam_*k*_*i*_&*k*_*j*_  (*i* ≠ *j*) represents damage in the form of reduced stiffness *k*_*i*_ and *k*_*j*_ at multiple sites, and 15% storey stiffness is reduced in this study.

**Figure 2:**
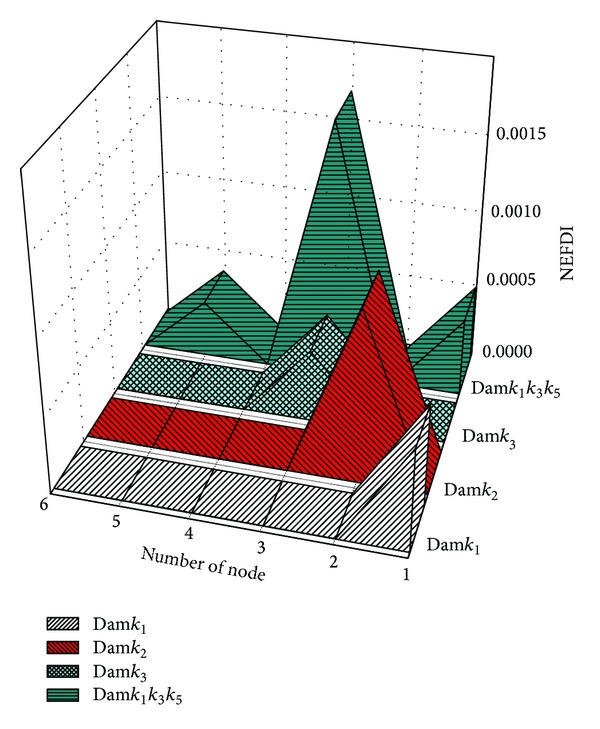
The identified damage locations: (Dam*k*_1_) damaged on 1st storey, (Dam*k*_2_) damaged on 2nd storey, (Dam*k*_3_) damaged on 3rd storey, and (Dam*k*_1_ *k*_3_ *k*_5_) damaged on 1st, 3rd, and 5th storey.

**Figure 3:**
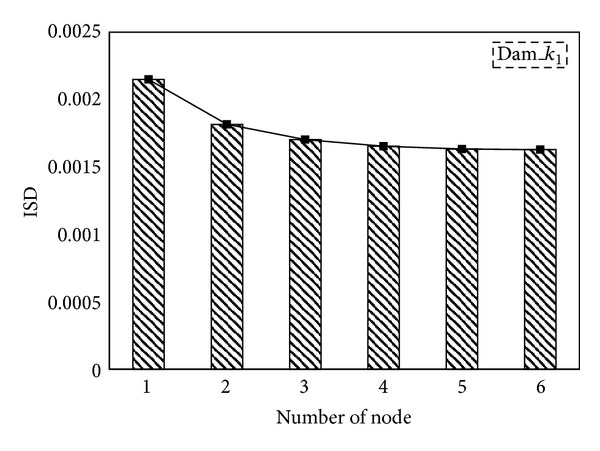
The *in situ* damage index, ISD_*i*_: damaged on 1st storey.

**Figure 4:**
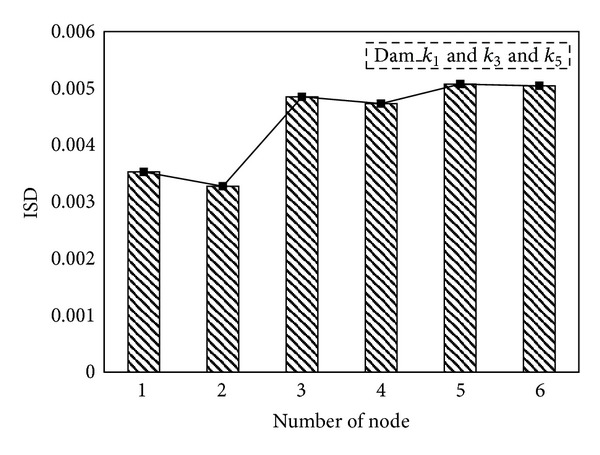
The *in situ* damage index, ISD_*i*_: damaged on 1st, 3rd, and 5th storey.

The locations of damage were determined using the NEFDI.  Figure 2 presents the identified damage locations in three cases of single instances of damage, Dam_*k*_1_, Dam_*k*_2_, and Dam_*k*_3_, and in one case of multiple instances of damage, Dam_*k*_1_ and *k*_3_ and *k*_5_. When damage occurred only to the *i*th storey, the positive value of the NEFDI that corresponds to the *i*th node greatly exceeded those that correspond to the other nodes.  Figure 3 illustrates the identified damage locations using (12). As you can see in the figure, the value of the ISD that corresponds to the first node greatly exceeded those that correspond to the other nodes. The result shows that the ISD can also indentify the first storey damage. However, after applying a multiple damage case, it becomes more difficult to locate the damage as shown in Figure 4. Accordingly, comparing to Figure 2, the proposed NEFDI is more practical in indentifying the multiple damage case.

**Figure 5:**
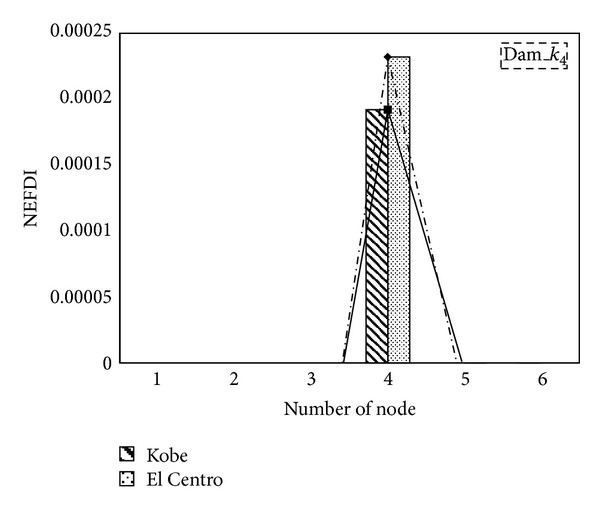
The NEFDI values of different nodes with different earthquake excitations.

The proposed index is confirmed to have located the damaged storey in cases of damage at a single site and at multiple sites. To demonstrate that the proposed index is independent of the responses to various input earthquakes and depends only on the structural properties, the Kobe and El Centro earthquakes were applied as input excitations.  Figure 5 presents the NEFDI values of various nodes. The NEFDI correctly locates the damage. The NEFDI value in El Centro earthquake case is higher than the Kobe case. It means that even the damage level is the same (15% storey stiffness is reduced in this case); the input energy will influence the value of NEFDI.

**Figure 6:**
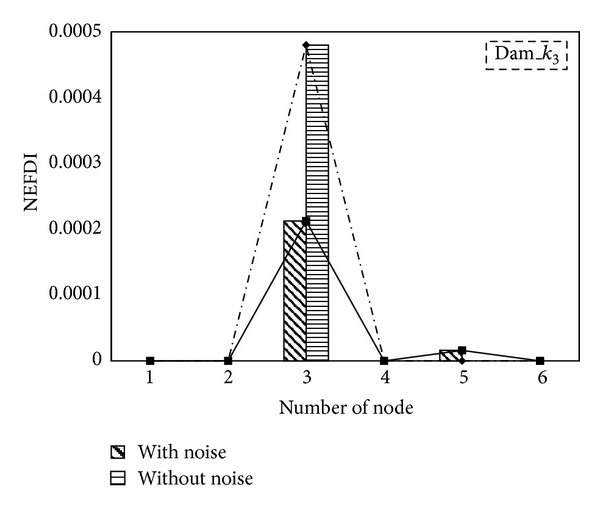
The effects of noise on NEFDI estimations.

To reflect the fact that measured data always contain noise, 5% white noise was added to the numerically simulated responses and input acceleration in the case of damage.  Figure 6 presents the effects of noise on the NEFDI estimations. Although noise affects NEFDI values, the NEFDI was still, correctly, the highest positive at node 3.

## 4. Development of NEFDI-Based Protocol in Imote2.NET-Based Wireless SHM System

**Figure 7:**
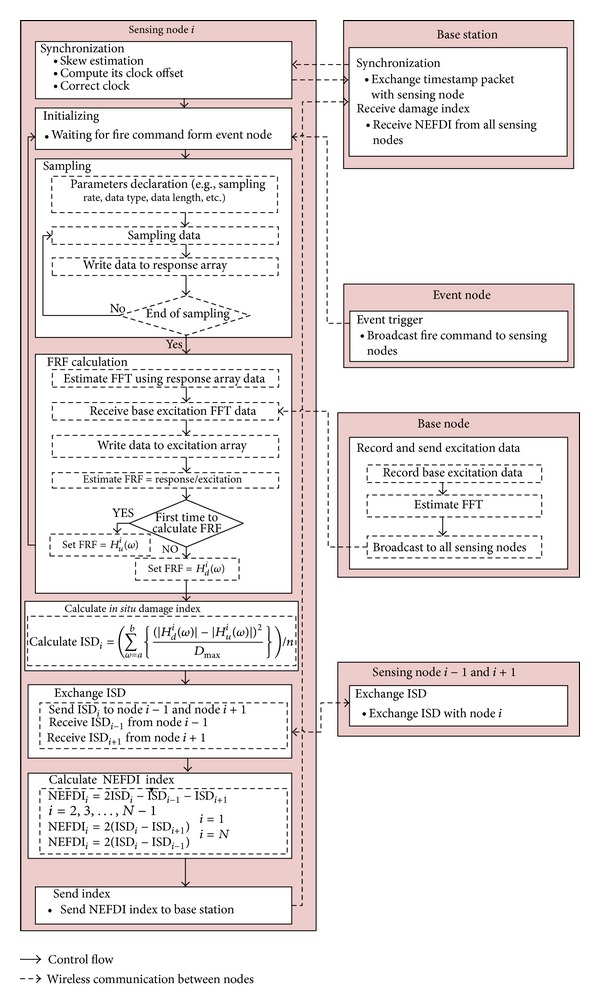
NEFDI-based protocol in the Imote2.NET SHM system.

An NEFDI-based protocol was developed and incorporated in the Imote2.NET-based wireless SHM system. This wireless SHM system was developed based on Imote2.NET, an advanced sensor platform that is compatible with the .NET Micro Framework.  Figure 7 presents the NEFDI protocol as applied with the Imote2.NET SHM system. Initially, the sending nodes and base station exchange timestamp packets to enable them to be synchronized by two-phase synchronization [29]. During the first phase, the proposed protocol models the skew in the clocks associated with all sensing nodes; each node is then skew-synchronized. Next, the skew is estimated by performing linear regression over multiple timestamp packets from the base station. Following synchronization, the sensing node waits for the fire command from the event node. When an earthquake occurs, the event node immediately broadcasts a packet with a fire command to the sensing nodes. Finally, the sampling procedure for all sensing nodes begins sampling data and writing them to a response array. Corresponding parameters, such as sampling rate, data type, and data length, are declared before sampling starts.

Following sampling, the FRF calculation is conducted. The base node then broadcasts base excitation data to all sensing nodes. The base excitation data are written to an excitation array. An FFT function then generates both response and excitation data in the frequency domain by applying the fast Fourier transformation procedure. FRF is then estimated and is locally stored. Initially, the FRF is estimated as a known undamaged state. Following each subsequent earthquake, the FRFs are referred to as a damaged state. After the undamaged and damaged FRFs have been obtained, the damage index, ISD_*i*_, can be estimated at all sensing nodes. The sensing nodes then exchange their ISDs with their neighbors to calculate the NEFDI. Finally, all sensing nodes send their NEFDIs to the base station to evaluate damage to the structure.

In the centralized scheme, data from all sensing nodes must be transmitted to the base station before the FRF is calculated. *N* · *N*_*p*_ transmissions are required to process all of these data, where *N*_*p*_ is the number of packets for transmitting all sensing data. The proposed protocol requires *N*_*f*_ + 3*N* transmissions only, where *N*_*f*_ denotes the number of packets that the base station must broadcast to all sensing nodes. Since *N*_*f*_ is associated with the number of FFT data, only half of all of the data are transferred. 3*N* is the number of ISD exchanges plus the number of NEFDI transmissions by *N* nodes. The total amount of transmitted data is much lower than in the conventional centralized protocol. As the number of nodes increases, the advantage of this protocol becomes considerable in terms of the power required for wireless communication. In this protocol, the base station node, event node, and base node are powered using a wall plug to keep them continuously awake. The sensing nodes are powered using a rechargeable battery with an energy-harvesting system. All sensing nodes are set to be in deep sleep mode and to wake up in turns. At least one sensing node must be awake on each floor to ensure measuring and others can be put into deep sleep mode (drawing 525 *μ*A) for a specified period.

## 5. Electromechanical-Impedance- (EMI-) Based Local Damage Detection Method

### 5.1. Fundamental of EMI-Based Model

**Figure 8:**
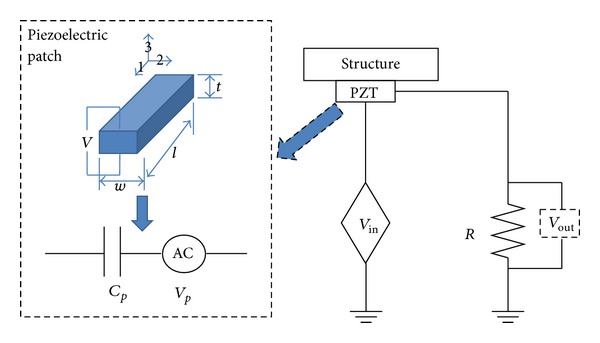
Diagram of PZT-structure bonded system.

The EMI-based damage detection approach exploits the EM coupling effect between active surface-bonded piezoelectric patches and the host structure. The literature [16, 23] shows that the EMI is related to the mechanical impedance of a host structure, enabling monitoring of the host structure by analyzing electrical impedance. A piezoelectric patch-structure bonded system can be modeled as a circuit system [30] (Figure 8). Herein, the piezoelectric patch was modeled as a capacitor (*C*_*p*_) and a self-sensing-actuation voltage source (*V*_*p*_) caused by input voltage (*V*_in_). The output voltage, which was coupled with *V*_*p*_ and *V*_in_, can be expressed as

(15) $$\begin{array}{c} V_{\text{out}}\left(\omega \right)=\frac{Z_{R}\left(\omega \right)}{Z_{R}\left(\omega \right)+Z_{p}\left(\omega \right)}\left(V_{\text{in}}\left(\omega \right)+V_{p}\left(\omega \right)\right), \end{array}$$

where *Z*_*R*_ is the electrical impedance of the resistor, and *Z*_*p*_ is the electrical impedance of the piezoelectric patch. The electrical impedance of the piezoelectric patch can then be expressed as

(16) $$\begin{array}{c} Z_{p}\left(\omega \right)=Z_{R}\left(\omega \right)\left(\frac{V_{\text{in}}\left(\omega \right)+V_{p}\left(\omega \right)}{V_{\text{out}}\left(\omega \right)}-1\right). \end{array}$$

Studies [20, 30, 31] demonstrate that the self-sensing-actuation voltage source is related to mass, damping, and stiffness of the host structure. Equation (16) indicates that *Z*_*p*_ is related to structural damage to the host structure. A piezoelectric patch-structure bonded system can be further modeled as an electromechanical admittance (EMA) model (inverse of EMI). The electromechanical admittance model can be expressed as

(17) Y(ω)=jωwlt((ε−33T−d312Y^E) +Zpm(ω)Zpm(ω)+Zs(ω)d312Y^E(tan(k^l)k^l)),

where *w*, *l*, and *t* are the width, length, and thickness of the piezoelectric patch, respectively; ${\hat{Y}}_{}^{E}$ is the complex Young's modulus of the piezoelectric patch in zero electric field; ${\overset{-}{\varepsilon }}_{33}^{T}$ is the complex dielectric constant of the piezoelectric patch; *d*_31_^2^ is the coupling constant of the piezoelectric patch in the *x* direction at zero stress; $\hat{k}=\omega \sqrt{\rho /{\hat{Y}}_{}^{E}}$ is the wave number, which is related to density *ρ*, ${\hat{Y}}_{}^{E}$, and excitation frequency, and *Z*_*pm*_ and *Z*_*s*_ are the mechanical impedance of the piezoelectric patch and the host structure, respectively. The mechanical impedance of an SDOF structural system is given by

(18) $$\begin{array}{c} Z_{s}\left(\omega \right)=\frac{F_{0}}{\dot{\nu }}=\left|Z_{s}\left(\omega \right)\right|e^{i\theta } \end{array}$$

in which

(19) $$\begin{array}{c} \left|Z_{s}\left(\omega \right)\right|=\sqrt{c^{2}+\frac{\left(m\omega^{2}-k\right)}{\omega^{2}}}, \end{array}$$

where *F*_0_ is a harmonic excitation force at angular frequency *ω*; $\dot{\nu }$ is the velocity response in the frequency domain, and *m*, *c*, and *k* are the mass, damping, and stiffness of the host structure, respectively.

Equation (17) indicates that EMI (or EMA) is directly related to the mechanical impedance of a host structure. According to (18) and (19), damage to a structure alters its mechanical impedance. Therefore, based on an assumption of a constant mechanical impedance of the piezoelectric patch, changes in the EMI signal are related to damage to the structure of the host.

Although the EMI provides a method for qualitatively detecting structural damage, a quantitative approach is still required. The conventional approach for quantifying damage uses root mean square deviation (RMSD), which is a simple statistical algorithm that is based on frequency-by-frequency comparisons [21, 30, 32]. The RMSD is defined as

(20) $$\begin{array}{c} \text{RMSD}=\sqrt{\frac{\sum_{i=fs}^{fe}{\left(I_{i}^{D}-I_{i}^{U}\right)}^{2}}{\sum_{i=fs}^{fe}{\left(I_{i}^{U}\right)}^{2}}}\times 100, \end{array}$$

where *fs* and *fe* are the start and end frequencies of the impedance, respectively. *I*_*i*_^*U*^ and *I*_*i*_^*D*^ are the real parts of the impedance of piezoelectric patch at the *i*th frequency of a particular structure in undamaged and damaged states, respectively. In an RMSD damage metric chart, a greater numerical value of the metric corresponds to a larger difference between the baseline reading and the subsequent reading, indicating greater damage to the host structure.

### 5.2. Measurement of Piezoelectric Impedance

Piezoelectric impedance is typically measured using an impedance analyzer. However, the disadvantages of this device include its large size, high cost, and lack of portability. Peairs et al. [33] developed a device that combines a digital signal analyzer with an FFT function for measuring and recording the electric impedance of a PZT. The advantages of their impedance-measuring device are its low cost and small size.

Analog Device, Inc., recently introduced the AD5933 Micro Electronic Mechanical System (MEMS), a single-chip impedance measurement device. This high-precision device combines an on-board frequency generator with a 12-bit, 1 MSPS analog-to-digital converter (ADC). The frequency generator allows excitation of external complex impedance at a known frequency. The response impedance signal is sampled by the on-board ADC and a DSP with discrete Fourier transformation (DFT). The impedance and relative phase thereof at each frequency point are easily determined from calibrated real (R) and imaginary (I) data that are obtained by performing off-chip calculations using real and imaginary register contents that are read from the serial I2C interface.

## 6. Experimental Study

**Figure 9:**
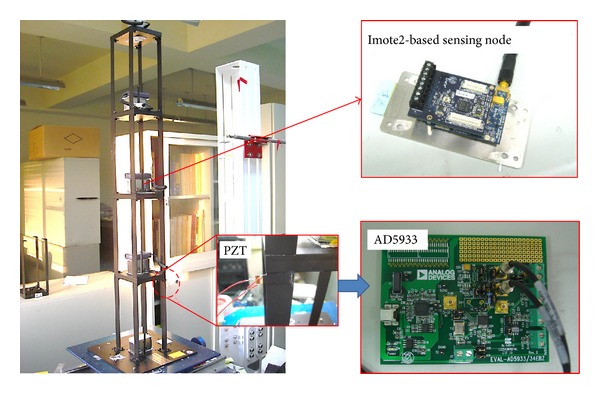
Experimental setup for wireless SHM system and impedance measurement device.

**Figure 10:**
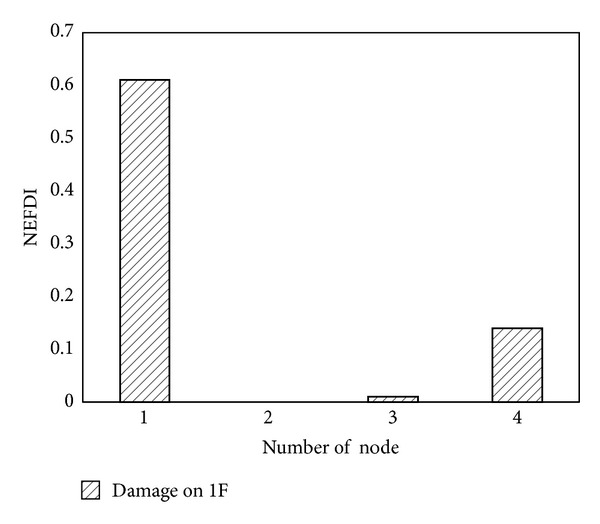
The NEFDI: damage on the 1st floor.

In this experimental study, the proposed damage detection approach was firstly applied to a four-storey steel frame model (Figure 9) on a Quanser shaking table. Each floor weighed approximately 4 kg; each column had a cross-sectional area of 40 mm^2^ and a height of 210 mm. A wireless sensing node was deployed at the center of each floor. Four PZT patches were attached to each joint of the first-floor columns. After the various baseline impedance signatures and acceleration had been measured, damage was simulated by completely loosening two bolts at joint 4 on the first floor. Integrated global-local damage detection approach is applied to locate the damage in this experimental study. The first stage is the global approach which detects changes in the characteristics of the overall structure. Then, in the second stage, the damage is identified in detail and its approximate location is determined. In this experimental building structure, global SHM NEFDI index can yield a rough estimate of the location of a damaged floor. Local SHM then checks in detail the components of the structure of the building using EMI-based method. As shown in Figure 10, the positive value of the NEFDI that corresponds to the first node greatly exceeded those that correspond to the other nodes. As a result, the first floor damage can be identified (Figure 10).

**Figure 11:**
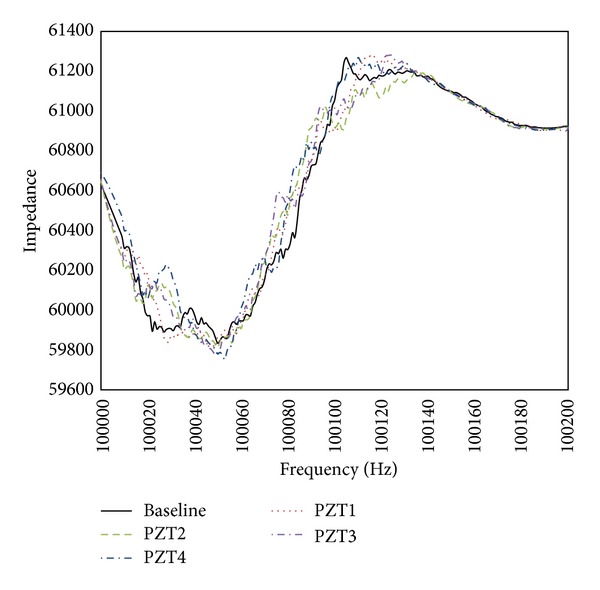
A baseline of undamaged state and four damaged impedance curves.

**Figure 12:**
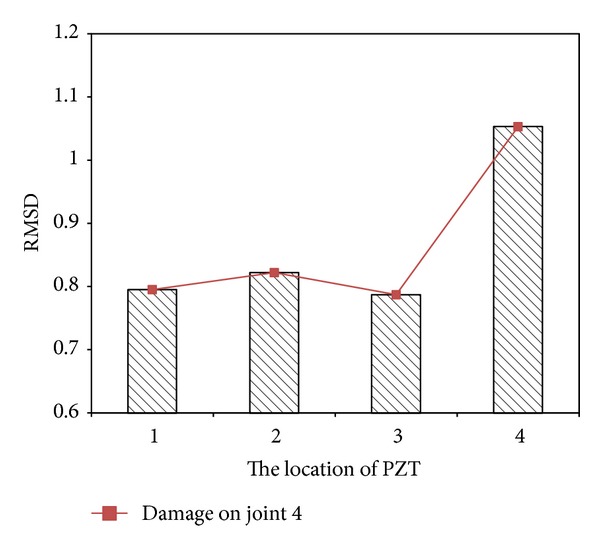
A damage metric chart of RMSD for damage on joint 4.

**Figure 13:**
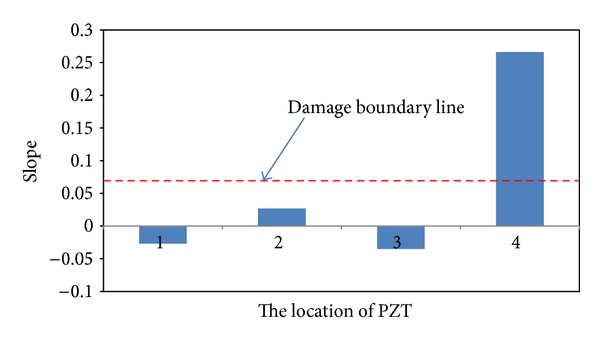
A damage metric chart of RMSD's slope for damage on joint 4.

Next step is applying EMI-based method to locate the loosening two bolts.  Figure 11 presents the baseline undamaged state and four measurements of the real part of impedance of the PZT at joints 1–4. It is hard to determine the loosening two bolts by only utilizing these impedance curves of the PZT at joints 1–4. Hence, (20) is applied to obtain RMSD index for locating the damage.  Figure 12 displays a damage metric chart, based on RMSD. It is constructed to identify the position to locate local damage to the structure. As shown in Figure 12, the positive value of the RMSD that corresponds to joint 4 exceeded those that correspond to the other joints.  Figure 12 thus demonstrates that joint 4 exhibited the most damage. However, the difference of RMSD between joint 4 and other joints is not great enough. To increase the difference, a slop of RMSD is applied.  Figure 13 illustrates a damage metric chart of RMSD's slope. Obviously, the RMSD that corresponds to joint 4 significantly exceeded those that correspond to the other joints. As a result, a damage boundary line can be set to 25% of the most significant RMSD's slope to distinguish the damaged and undamaged state.

**Figure 14:**
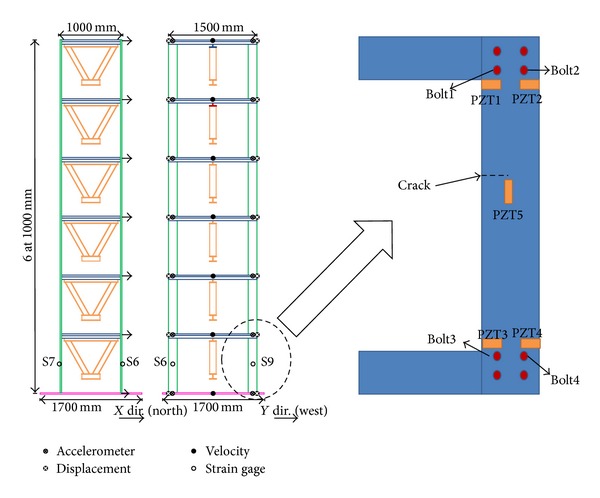
Experimental setup of the test structure.

The following experimental study was performed to confirm the practicality of the proposed embedded damage detection approach with the proposed Imote2.NET-based wireless SHM system on a steel frame model. The model was a (1/4)-scale six-storey steel structure that was designed by the NCREE, Taiwan.  Figure 14 presents the test structure. Its floors, beams, and columns were connected to each other using bolts. Various measurement sensors, including triaxial accelerometers, an LVDT, and velocity sensors, were deployed. An Imote2-based wireless structural health monitoring system was utilized for sensing, logging, storing, processing, and analyzing the data that were recorded from the sensors. Damage was simulated by reducing the width of the middle of each column on the first floor. All experiments involved excitation using a 100 gal El Centro earthquake input on a shaking table at NCREE.

In a WSN, some packets are lost during RF communication. For instance, packets may collide if multiple nodes attempt to send packets to the base station simultaneously. Packet loss may thus result in the incompleteness of measurement signals. The successful packet received rate (SPRR) was calculated to assess the reliability of data transmission among nodes. SPRR is defined as the number of successfully received packets divided by the number of packets sent.

**Figure 15:**
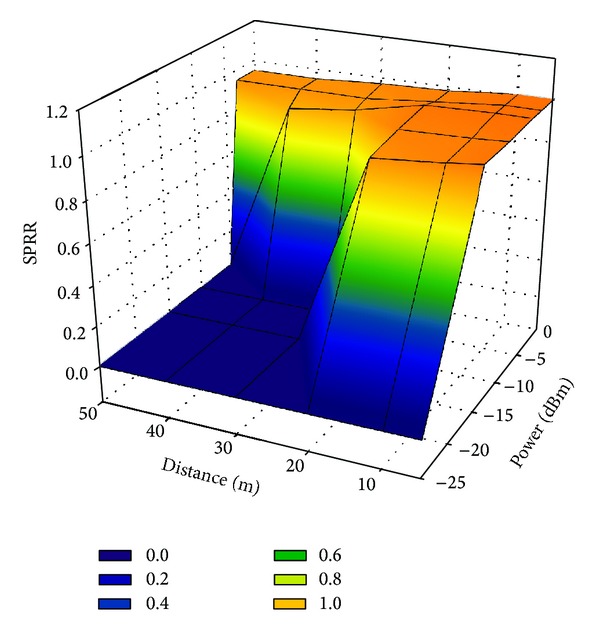
Successful packet received rate (SPRR) to RF Power and distance.

In practice, sensing nodes send a fixed number of packets to a base station. The base station can therefore calculate the SPRR according to the number of sent and received packets. The experimental results herein reveal that the average SPRR is almost 99% over 10, 30, 60, or 120 minutes. This result reveals that data loss was less than 1% even with an extended measuring period. The SPRR was used to evaluate the intervals between the sending of packets. The wireless communication test in this investigation shows that a packet sending interval of 20 ms yielded an SPRR of approaching 1.0, but the SPRR decreased declined as the packet sending interval decreased.  Figure 15 plots the relationship among RF power, distance, and the SPRR. Clearly, transmission range depends on RF power. A higher power output not only increases range but also increases power consumed.

Therefore, Figure 15 is a useful reference for deploying sensor nodes in a structure. Each node can be allocated a different transmission power to optimize power consumption for various distances. In the experimental study herein, the greatest distance between sensing node and receiving node was 20 m. Accordingly, the RF power was set to −15 dbm to reduce power consumption. Also, the SPRR will decrease dramatically if distance exceeds a giver range. Subsequently, based on this figure, we can know where to set up a base station for receiving the data from sensing nodes.

Following the preliminary investigation, the Imote2-based wireless structural health monitoring system with NEFDI protocol was adopted on a steel frame model. The measurement period was 60 s, during which data were sampled at 200 Hz. Along with the novel protocol, a time-scheduling data transmission procedure was applied to transfer raw data to the base station. Initially, the sensing nodes wait until sending-delay time equals (total packet sending time) ∗ (node ID). For instance, if the node ID is 0, then sensing node 0 transmits a packet immediately, since the sending delay is zero. By this procedure, each node sequentially sends its data to the base station with its particular sending delay, preventing the packet collision.

**Figure 16:**
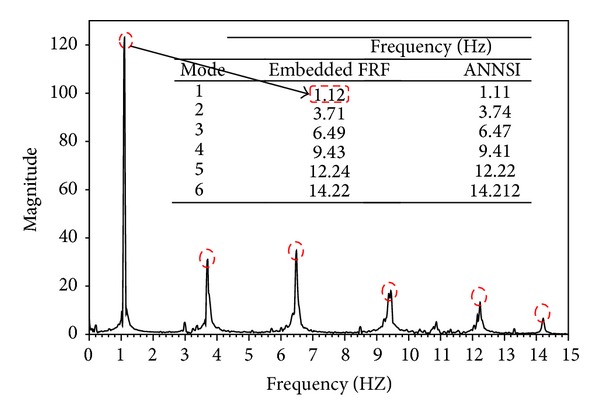
Comparisons of modal parameters identified by embedded FRF and ANNSI.

Figure 16 compares the modal parameters that were evaluated using the embedded FRF and ANN-based system identification (ANNSI) [34] approach with the raw data that were measured by Imote2.NET-based sensing nodes. Comparisons revealed that modal parameters identified by embedded FRF and ANNSI are approximated. They confirm the effectiveness of the proposed protocol. Accordingly, the proposed approach is more practical in embedded application than the ANN-based method. ANN-based system identification needs high performance computation hardware that is not suitable for resources limited WSN application.

Although the proposed protocol is reasonable for wireless-based SHM, the performance of embedded processing must be evaluated.  Table 1 presents the processing time of the embedded FFT and FRF for various amounts of data. In practice, the number of FFT data points is typically 1,024 or 2,048. The average processing times for FFT and FRF with 1,024 points are 0.193 s and 0.064 s, respectively. Obviously, when the data length is increased from 512 to 4096, the processing times for FFT is increased to ten times. The processing of FFT is always resource constrained need carefully to choose the hardware and software implementation. The same FFT code was implemented in a 200 MHz ARM processor-based .NET Micro Framework device. The average processing times for FFT and FRF with 1,024 points were then 0.8 s and 0.25 s, respectively. Clearly, the Imote2 with the XScale processor and enhanced DSP core is sufficiently powerful to process data in SHM.

**Figure 17:**
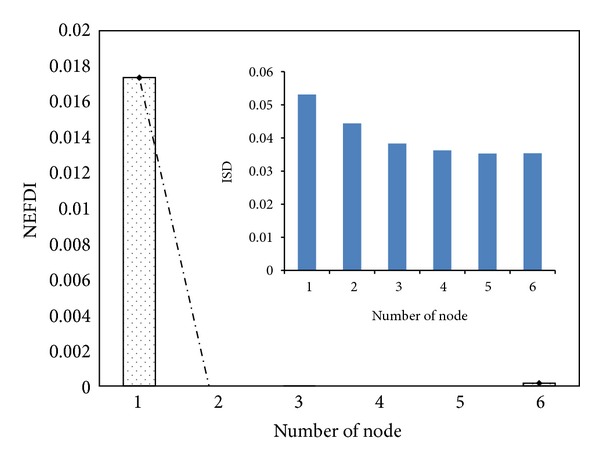
The identified damage locations: damaged on 1st Floor.

Numerically simulated responses demonstrate that the proposed NEFDI successfully located damage. Integrated global-local damage detection approach is applied to locate the damage in this experimental study. The first stage is the global approach which detects changes in the characteristics of the overall structure. Then, in the second stage, the damage is identified in detail and its approximate location is determined. First, NEFDI was applied to locate damage in an experimental frame, as presented in Figure 17. The width of the middle each column was reduced by 7.5 cm on the first floor. The highest corresponding positive NEFDI value revealed that the damage was most severe on the first floor. Figure 17 shows the identified damage locations using (12). As shown in the figure, the value of the ISD that corresponds to the first node exceeded those that correspond to the other nodes. The result shows that the ISD can also indentify the first storey damage. However, NEFDI can decrease the influence in undamaged node, to break out the damage state.

**Figure 18:**
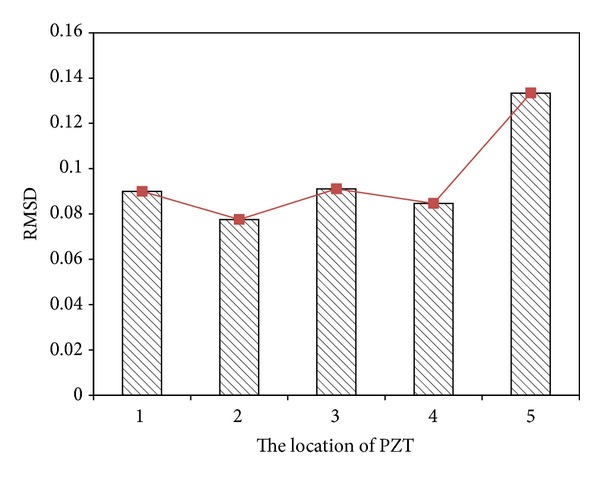
A damage metric chart of RMSD for damage.

**Figure 19:**
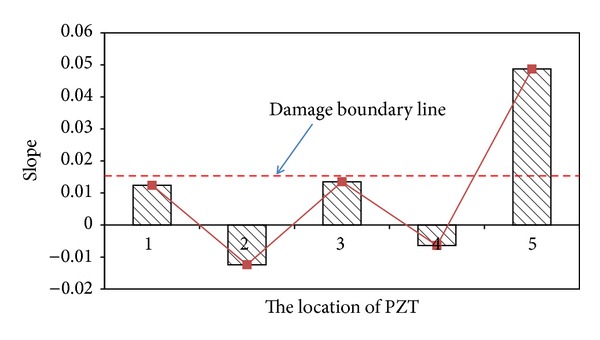
A damage metric chart of RMSD's slope for damage.

In the first stage, the damage was identified in first floor using NEFDI. Next step is to identify the location of damaged column. Therefore, four PZT patches were attached to each joint of the first-floor columns and one PZT patch was attached near the damage place in the middle of the columns. EMI-based method was used to obtain the baseline undamaged state and five measurements of the real part of impedance of the PZTs. A damage metric chart, based on RMSD, was then constructed to identify local damage.  Figure 18 shows that the most severe damage was at location 5. It proves that the damage is in the middle of the column.  Figure 19 illustrates a damage metric chart of RMSD's slope. Obviously, the RMSD that corresponds to joint 5 significantly exceeded those that correspond to the other joints. A damage boundary line can also be set to 25% of the most significant RMSD's slope to distinguish the damaged and undamaged state. Whereas the impedance method cannot precisely quantify damage, the damage metric chart provides useful quantitative information.

## 7. Concluding Remarks

An integrated global-local damage detection approach was developed for locating damage to building structures. An embedded damage detection approach with a novel embedded FRF damage index (NEFDI) is proposed to detect global damage. Local damage was then identified using the EMI-based method. In this work, electromechanical impedance was measured using single-chip impedance measurement devices.

An NEFDI-based protocol was presented and experimentally implemented in the proposed Imote2.NET-based wireless SHM system. The proposed .NET Micro Framework-based software system supported the efficient development of individual SHM applications. A useful figure was established as a reference for the allocation of transmission power to optimize power consumption for various distances between sensing node and receiving node. The processing times of the embedded FFT and FRF for various data lengths were evaluated, revealing that Imote2 with the XScale processor and enhanced DSP core is sufficiently powerful for processing data obtained by SHM.

Damage was located using the NEFDI. Numerical and experimental studies confirmed the effectiveness of the proposed approach. Experimental analysis of the integrated global-local SHM approach reveals that, after NEFDI indicates the approximate location of a damaged area, the EMI-based damage detection approach can check the specific components of the building structure. A damage metric chart, based on RMSD, is constructed to locate local damage to the structure.

## Figures and Tables

**Table 1:** The processing time of embedded FFT and FRF for different data lengths.

	Data length			
	512	1024	2048	4096
FFT	0.089 s	0.193 s	0.442 s	0.970 s
FRF	0.032 s	0.064 s	0.130 s	0.259 s

Total	0.121 s	0.256 s	0.572 s	1.229 s
